# Effects of a human amniotic membrane extract on ARPE-19 cells

**DOI:** 10.1007/s11033-024-09647-7

**Published:** 2024-06-14

**Authors:** Matteo Lulli, Ruggero Tartaro, Laura Papucci, Lucia Magnelli, Indu Pal Kaur, Tomaso Caporossi, Stanislao Rizzo, Antonella Mannini, Fabrizio Giansanti, Nicola Schiavone

**Affiliations:** 1 Department of Experimental and Clinical Biomedical Sciences ‘Mario Serio’, University of Florence, Florence, Italy; 2 Department of NEUROFARBA, Ophthalmology, University of Florence, Careggi, Florence, Italy; 3 UGC-Centre of Advanced Study, University Institute of Pharmaceutical Sciences, Panjab University, Chandigarh, India; 4 Vitreoretinal Surgery Unit, Isola Tiberina Gemelli Isola Hospital, Rome, Italy; 5 Catholic University Sacro Cuore, Rome, Italy; 6 Department of Ophthalmology, Catholic University of Sacred-Heart Foundation “Policlinico Universitario A. Gemelli” IRCCS, Rome, Italy; 7 Department of Experimental and Clinical Medicine - Internal Medicine Section, University of Florence, Florence, Italy

**Keywords:** Human amniotic membrane, ARPE-19, PVR, Retina, Apoptosis, EMT

## Abstract

**Background:**

Human Amniotic Membrane (hAM) is endowed with several biological activities and might be considered an optimal tool in surgical treatment for different ophthalmic pathologies. We pioneered the surgical use of hAM to treat retinal pathologies such as macular holes, tears, and retinal detachments, and to overcome photoreceptor damage in age-related macular degeneration. Although hAM contributed to improved outcomes, the mechanisms of its effects are not yet fully understood. The characterization and explanation of the effects of hAM would allow the adoption of this new natural product in different retinal pathologies, operative contexts, and hAM formulations. At this end, we studied the properties of a hAM extract (hAME) on the ARPE-19 cells.

**Methods and results:**

A non-denaturing sonication-based technique was developed to obtain a suitable hAME. Viability, proliferation, apoptosis, oxidative stress, and epithelial-mesenchymal transition (EMT) were studied in hAME-treated ARPE-19 cells. The hAME was able to increase ARPE-19 cell viability even in the presence of oxidative stress (H_2_O_2_, TBHP). Moreover, hAME prevented the expression of EMT features, such as EMT-related proteins, fibrotic foci formation, and migration induced by different cytokines.

**Conclusions:**

Our results demonstrate that the hAME retains most of the properties observed in the whole tissue by others. The hAME, other than providing a manageable research tool, could represent a cost-effective and abundant drug to treat retinal pathologies in the future.

**Supplementary Information:**

The online version contains supplementary material available at 10.1007/s11033-024-09647-7.

## Introduction

The human amniotic membrane (hAM) is the tissue surrounding the embryo, composed of a stromal and a cellular component. An epithelial single-cell layer is placed on a stroma with embedded mesenchymal cells. The interest in the hAM’s unique properties and its widespread availability increases in the field of regenerative medicine [1, 2]. However, the hAM has been employed in anterior eye chamber surgery since 1940 to treat corneal wounds [3]. Although the hAM has a simple tissue architecture, its molecular composition is very rich in bioactive molecules [1, 2]. De-epithelized hAM may be an optimal substrate to grow retinal pigmented epithelial cells for possible use in allografts [4]. Recently, we successfully used hAM patches to surgically repair retinal defects such as macular holes and retinal tears [5, 6]. The hAM, when implanted inside the macular holes and retinal breaks, induced mechanical closure of these lesions accompanied by anatomical and partial functional recovery, due to the plug effect [5]. We postulated that a pro-regenerative action on the retinal layers might have played a role. However, the regenerative effect is difficult to document with the in vivo retinal imaging methods existing today, while studies on the biological activities of hAM on retinal cells in vitro are relatively scarce.

The retina is a multi-layered tissue, constituted by many different cell types. Among them, retinal pigmented epithelium (RPE) plays a pivotal structural and trophic role in sustaining photoreceptor homeostasis and functionality [7]. RPE cells are also one of the main cell types involved in side effects of retinal injury and surgery such as proliferative vitreoretinopathy (PVR). PVR is a common consequence of retinal detachment (RD), leading to impaired vision and poor surgery outcomes [8]. In RD, the RPE cells that disperse in the vitreous or are exposed to vitreal factors such as TGF-β2, TNF-α, TGF-β1, IL-1, IL-6, may undergo a conversion to a mesenchymal phenotype epithelial-to mesenchymal transition (EMT) [9, 10]. Transdifferentiated cells begin to produce adherent scar membranes which impair vision, impede retinal reattachment, and are difficult to remove without causing retinal tears [8]. Despite many promising studies, a treatment to effectively prevent PVR is yet to come. Anti-PVR experimental attempts are usually made with drugs capable of preventing EMT, sometimes producing undesired side effects on the retina itself [11]. Although hAM’s antiangiogenic, anti-inflammatory, and antifibrotic properties have been described [12, 13], there are few studies on the possible use of hAM to treat PVR and its effects on RPE cells. The work of He and coworkers [14] reports the anti-EMT properties of a hAM-purified component, namely the heavy chain-hyaluronic acid/pentraxin 3, which shows antifibrotic effects on the human ARPE-19, RPE-derived, cells. Another recent study indicates that the whole hAM did not show toxic or proliferative effects on ARPE-19 cells, while it significantly increased their viability [15].

Our work presents the effects on ARPE-19 cells of a hAM extract (hAME) obtained from the same hAM preparation we employed in retinal surgery. Our results add knowledge that may be fruitful in view of a future optimized use of different hAM-derived products.

## Materials and methods

### Preparation of hAME

Frozen fragments of surgical grade hAM were obtained from the Tissue Bank “Centro delle Cornee Piero Perelli” (Lucca, Italy). The hAM was prepared, stored, and collected with informed consent in agreement with the Italian Guidelines approved on September, 14th, 2016, by the National Transplant Centre. A hAME was produced with a sonication method on ice. Fragments of hAM, derived from two different donors (I and II), previously stored at -80 °C in glycerol solution as preservative, were washed 5 times in 10 mL PBS and weighed. hAM fragments were placed in PBS at a 1:1 ratio V/W and submitted to sonication (Branson Sonifier 150, Emerson, USA) at 20 W with 7–15 pulses for 10 s to obtain hAME. Total protein content was assessed by the Bradford method (Bradford reagent, Sigma-Aldrich). The minimal time and number of pulses ensuring the maximal yields in terms of total proteins were assessed (see Results section). Our protocol yields about 1–3 µg/µL total proteins. Protein extracts were aliquoted and stored at -20 °C until use.

### Cells

ARPE-19 cells, obtained from American Type Culture Collection (ATCC, USA), were cultured in high glucose (4.5 g/L) Dulbecco’s Modified Eagles Medium (DMEM, Euroclone, Italy) supplemented with 10% fetal bovine serum (FBS) (Euroclone, Italy), 2mM L-glutamine solution (Euroclone, Italy) and Penicillin-Streptomycin solution (10,000 units penicillin and 10 mg streptomycin/ml, Euroclone, Italy) in a humidified incubator at 37 °C in 5% CO_2_. ARPE-19 cells were passed weekly by trypsinization and used until passage 6.

#### Chemicals and treatments

H_2_O_2_ (Sigma-Aldrich, USA) and *tert*-Butyl hydroperoxide solution (TBHP) (Sigma-Aldrich, USA) were used as oxidative apoptotic stimuli. Recombinant tumor necrosis factor-alpha (TNF-α) and transforming growth factor-beta 2 (TGF-β2) were from Peprotech (USA); transforming growth factor-beta 1 (TGF-β1) was from Abcam (UK). In the experiments with ARPE-19 cells, TNF-α, TGF-β2, TGF-β1, TBHP, and H_2_O_2_ were added 30 min after hAME. Treatments were done for 24–48 h as indicated in the Results section.

#### Cell viability

Viability was analyzed by the Cell Proliferation Reagent WST-1 (Roche, Switzerland). Cells were plated at sub-confluence (4.0 × 10^3^ cells/well) in 96 multiwell culture dish plates, treated for 24 h, and stained according to the manufacturer’s instructions by substituting the culture media with phenol red-free medium, added with WST-1 reagent. The plates were read by a multiple plate reader (Biorad, model 550, USA). Quantification of live and dead cells was performed by a Cytosmart apparatus (Corning, USA), using trypan blue staining (Merck, USA).

### Western blot analysis

Aliquots of 50 µg of ARPE-19 whole-cell lysates, obtained as previously described [16], were subjected to Western blotting. Protein extracts were separated by SDS-PAGE (Thermo-Fisher, USA) and transferred onto nitrocellulose membranes (Bio-Rad, USA). Membranes were incubated in Odyssey Blocking Solution (Millipore, Italy) for 1 h at room temperature. Membranes were then incubated overnight at 4 °C with the primary antibody (all primary antibodies were used diluted 1:1,000 in a mix of 1:1 Odyssey Blocking Solution and PBS-Tween 0.1%), washed with PBS-Tween 0.1% solution, and probed with the secondary IRDye antibody according to the manufacturer’s instructions (secondary antibody diluted 1:12,000). The primary antibodies were: ZO-1 (D7012, Cell signaling, USA), β-actin (sc-47,778, from Santa Cruz Biotechnology, USA), N-Cadherin (N-Cad) (4061P Cell signaling, USA). The protein bands were analyzed by the Odyssey Infrared Imaging System (LI-COR Bioscience, USA), using the Odyssey software for protein quantification.

### Cell motility

Cell motility was assessed using a Culture-Insert 4 Well (Ibidi, Germany). Briefly, 1.0 × 10^5^ cells were seeded in the wells of a culture insert located at the center of a 35 mm culture dish. After 24 h the culture insert was removed to reveal the wound gap of 500 μm. Then, cells were washed with PBS to remove floating cells, and fresh medium with 0.5% FBS, TGF-β1 (5 ng/mL), and hAME (50 µg/mL), was added. Pictures were taken at regular intervals until 48 h using a 10x phase contrast objective (Nikon, The Netherlands). The cell-free area was measured using ImageJ software via MRI wound healing plugin [17].

### Flow cytometry analysis of apoptosis

Cells were seeded (2.0 × 10^5^) in 35 mm Petri dishes. After 24 h cells were treated. After 48 h cells were detached by trypsin solution (Euroclone, Italy) and harvested with supernatants, centrifuged, resuspended in 500 µL of 1x Annexin Binding Buffer (BD Biosciences, USA), and incubated with 5 µL of Annexin V-FITC (BV421 Annexin V, BD Biosciences, USA) and 5 µL of and 7-AAD (BD Biosciences, USA) at room temperature for 30 min in the dark. Cells were then analyzed with a BD FACS Canto II (BD Biosciences, USA). Data were analyzed with Diva software (BD Biosciences, USA). A minimum of 10,000 events were collected.

### EMT-associated fibrotic deposit (EAFD) assay

EAFDs were analyzed as previously described [18]. Briefly, ARPE-19 cells were placed on 12-well plates at a density of 5.0 × 10^4^ cells/well. After 72 h, cells were washed twice with culture media without serum, and then cultured for 96 h without serum in the presence of TNF-α (10 ng/mL), TGF-β2 (5 ng/mL), or hAME. The cells were fixed with methanol for 5 min, air-dried at room temperature for 5 min, and stained with Giemsa solution (Merck, USA) for 15 min. Pictures of multiple fields were taken with a light microscope equipped with a camera and the area occupied by fibrotic deposits was quantified by the ImageJ software [17]: the contrast of the black and white images is increased, the area of the darker regions corresponding to the foci is drawn manually and the relevant % area is calculated automatically. Five fields for each experiment were analyzed.

### Statistics

Experiments were repeated at least three times. Results are expressed as means ± SD. Multiple comparisons were performed by the Student’s *t*-test or One-way or Two-way ANOVA using GraphPad Prism. Statistical significance was accepted at *p* < 0.05.

## Results

### hAME increases ARPE-19 cell viability

**Fig. 1:**
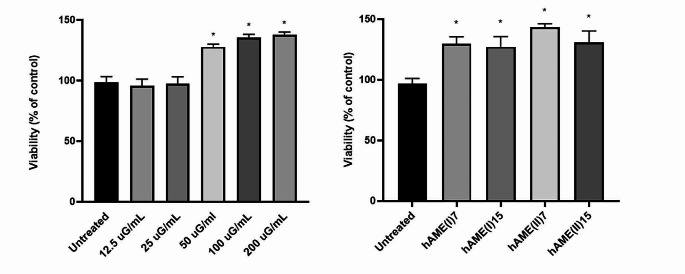
Effect of hAME on ARPE-19 cell viability. (**a**) with increasing concentration of hAME (from donor I) (12.5–200 µg/mL), obtained by 7 ultrasound pulses for 10 s on ice; (**b**) with two hAMEs from different donors (I and II), obtained by different numbers of ultrasound pulses (7 and 15 for 10 s on ice) (see Methods Section); ARPE-19 cells were treated for 24 h with hAME (50 µg/ml). * *p* < 0.05 vs. untreated

Based on the protocol used for preparation and storage, hAM fragments are considered to be non-viable. We preliminary observed that culturing ARPE-19 cells in the presence of a fragment of hAM (2 × 2 mm) for 24 h, determined a clear increment in cell number (data not shown). We consequently have been prompted to further characterize the properties of the tissue. In order to obtain reproducibility and manageability, we used the hAM in the form of an extract. ARPE-19 cells were treated with five different concentrations of hAME (12.5, 25, 50, 100, 200 µg/mL), and cell viability was assessed 24 h after treatment (Fig. 1a). Treatment with 50 µg/mL maximally increased cell viability and was chosen for all the subsequent experiments. Also, hAMEs arising from hAM fragments from different donors gave no significant differences and were used interchangeably (Fig. 1b). Increasing the number of sonicator pulses (from 7 to 15) did not modify the effect on viability (Fig. 1b).

### hAME increases the proliferation of ARPE-19 cells

**Fig. 2:**
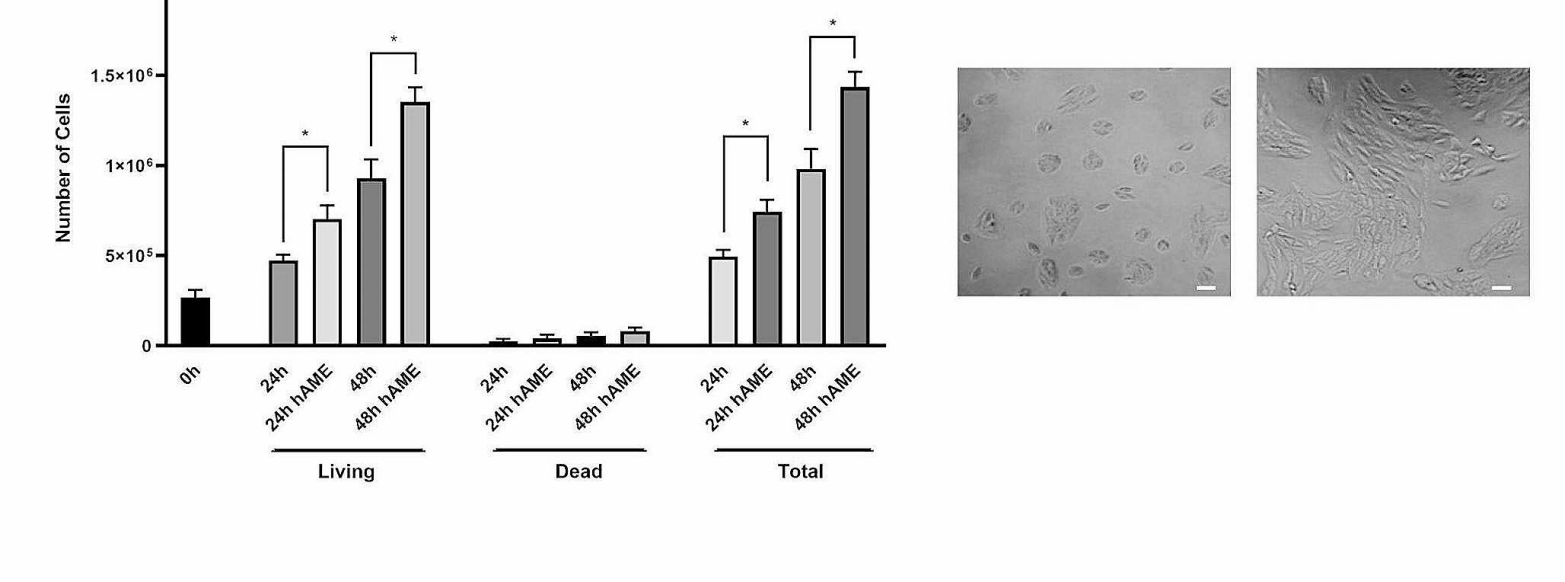
Effect of hAME on ARPE-19 cell proliferation. (**a**) Quantification of total living cells treated for 24–48 h with hAME (50 µg/mL) via trypan blue assay. (**b, c**) Representative microscopic images of ARPE-19 cells not treated or treated with hAME for 48 h, respectively (scale bar: 100 μm, 20X magnification). * *p* < 0.05 vs. 24 or 48 h

An increase in viability can result from different causes such as metabolic activity, proliferation, and apoptosis. In order to assess if the increase in viability was due to cell proliferation we counted the viable cell numbers at different time points (24 h and 48 h) using the trypan blue to distinguish between total living and dead cells at each time points. Cells were counted at 0, 24, and 48 h of hAME treatment (Fig. 2a-c). The number of living cells increased by about 30% in hAME-treated cells at both time points with respect to the untreated cells. Dead cells increased slightly and proportionally to the increase in cell proliferation albeit not significatively (Fig. 2a). On the whole, the results shown in Figs. 1 and 2 demonstrated that treatment with hAME determined an increase in ARPE-19 cell viability with concomitant induction of proliferation.

### hAME counteracts the effect of the oxidative stress on ARPE-19 cell viability

**Fig. 3:**
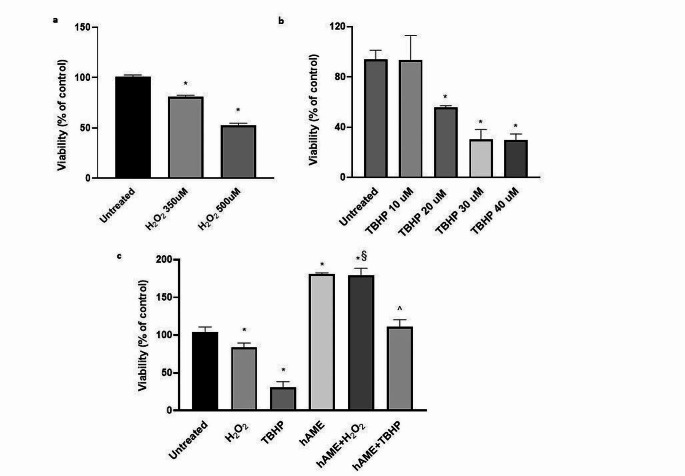
Effect of hAME on ARPE-19 cells exposed for 24 h to oxidative stress stimuli. Evaluation of ARPE-19 viability in the presence of (**a**) increasing concentration of H_2_O_2_; (**b**) increasing concentration of TBHP; (**c**) treatment with 350 µM H_2_O_2_ and 30 µM TBHP in combination with 50 µg/mL hAME. * *p* < 0.05 vs. untreated; § *p* < 0.05 vs. H_2_O_2_; ^ *p* < 0.05 vs. TBHP

Oxidative stress was induced in ARPE-19 cells by two different stimuli, H_2_O_2_ and TBHP, for 24 h. We performed dose-effect experiments and chose the concentration of H_2_O_2_ and TBHP that produced a 20% and 70% reduction in cell viability, i.e. 350 µM for H_2_O_2_ and 30 µM for TBHP, respectively (Fig. 3a-b). Figure 3c shows that treatment of ARPE-19 cells with hAME severely abolished the reduction of viability determined by H_2_O_2_ or TBHP.

### hAME does not protect ARPE-19 cells from oxidative stress-induced apoptosis

**Fig. 4:**
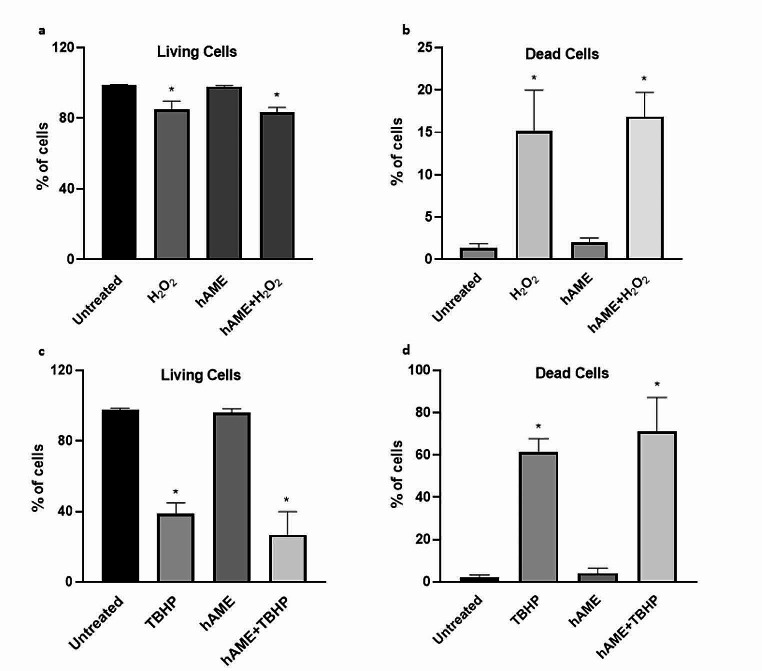
Effect of hAME on ARPE-19 cell apoptosis. (**a**) Dot plots of Annexin-V/7AAD evaluation via FACS analysis of cells treated with H_2_O_2_ (**a**) living and (**b**) dead cells or TBHP (**c**) living and (**d**) dead cells. * *p* < 0.05 vs. untreated

In order to assess if the hAME protects ARPE-19 cells from apoptosis induced by oxidative stress, FACS experiments were performed on ARPE-19 cells treated with hAME and exposed for 24 h to H_2_O_2_ and TBHP, at the same concentrations used in viability experiments (Fig. 4a-d and S1). The treatments with the two oxidants resulted mostly in early and late apoptotic death. Treatment with hAME did not protect ARPE-19 cells from apoptosis induced by H_2_O_2_ and TBHP.

### hAME inhibits EMT in ARPE-19 cells

**Fig. 5:**
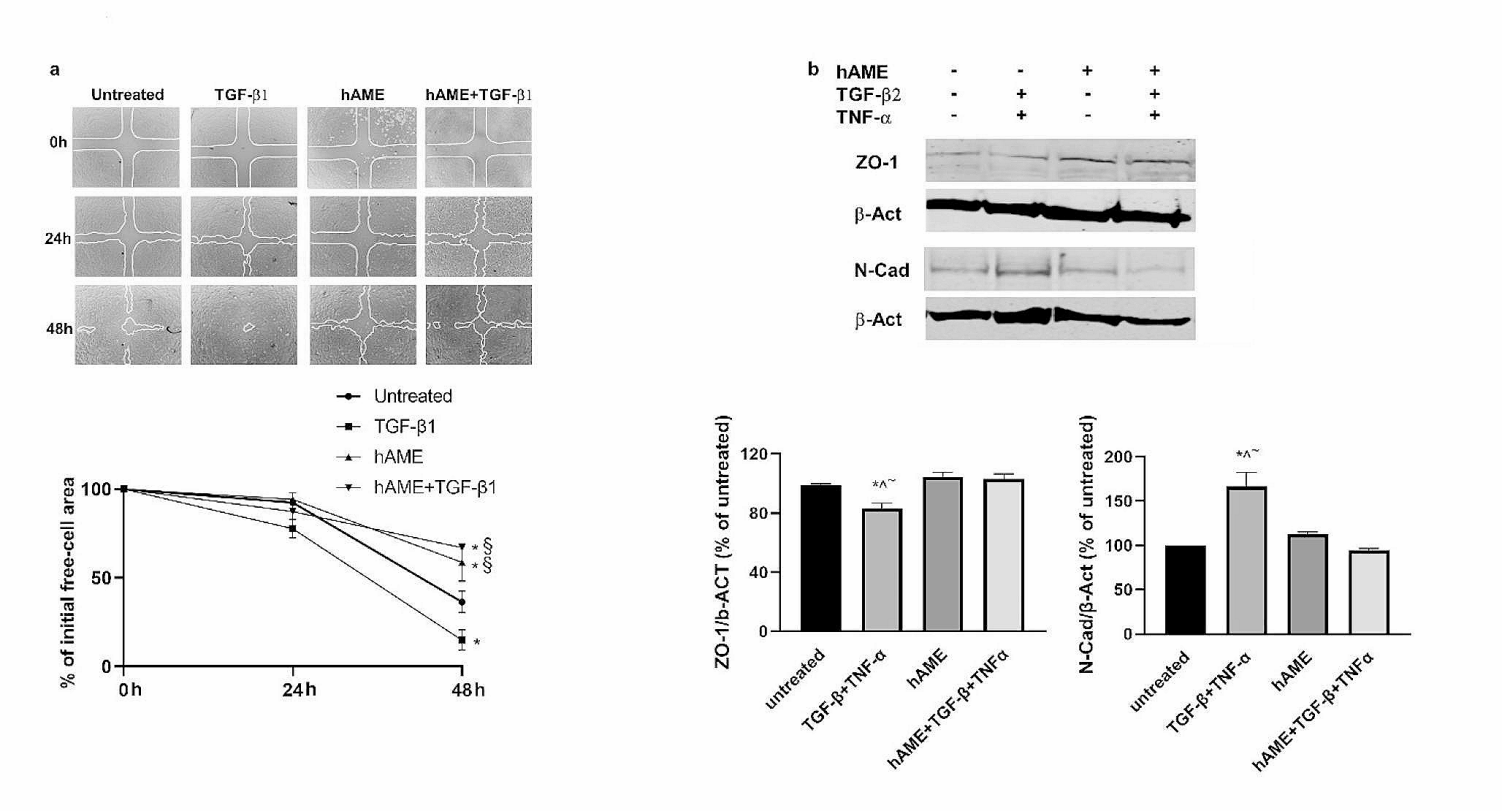
Effect of hAME on EMT-related traits of ARPE-19 cells treated with hAME in the presence or not of TGF-β1 or TNF-α + TGF-β2. (**a**) Motility of ARPE-19 was evaluated by wound healing assay and evaluation of the % free-cell area. (**b**) Western blot and relevant quantitative proteins in ARPE-19 cells. * *p* < 0.05 vs. untreated; ^ *p* < 0.05 vs. hAME; § *p* < 0.05 vs. TGF-β1; ~ *p* < 0.05 vs. hAME + TGF-β + TNF-α

First, a wound healing assay was employed to evaluate cell motility. ARPE-19 cells were seeded at confluence and treated for 24 and 48 h with hAME and/or TGF-β1, a known inducer of ARPE-19 cell motility [19]. Results shown in Fig. 5a reveal that the propensity to migrate was inhibited in cells treated with hAME by about 30% and 50% with respect to the untreated and TGF-β1 treated controls, respectively.

Moreover, protein expression of epithelial (Zonula occludens protein 1, ZO-1) and mesenchymal (N-Cad) markers was evaluated (Fig. 5b). Cells were cultivated in serum free medium treated for 24 h with TNF-α + TGF-β2, which reduced the expression of ZO-1 while increasing N-Cad. Treatment with hAME alone did not determine a significant alteration of both ZO-1 and N-Cad. Instead, hAME treatment was able to significantly counteract TNF-α + TGF-β2 modulation of ZO-1 and N-Cad.

**Fig. 6:**
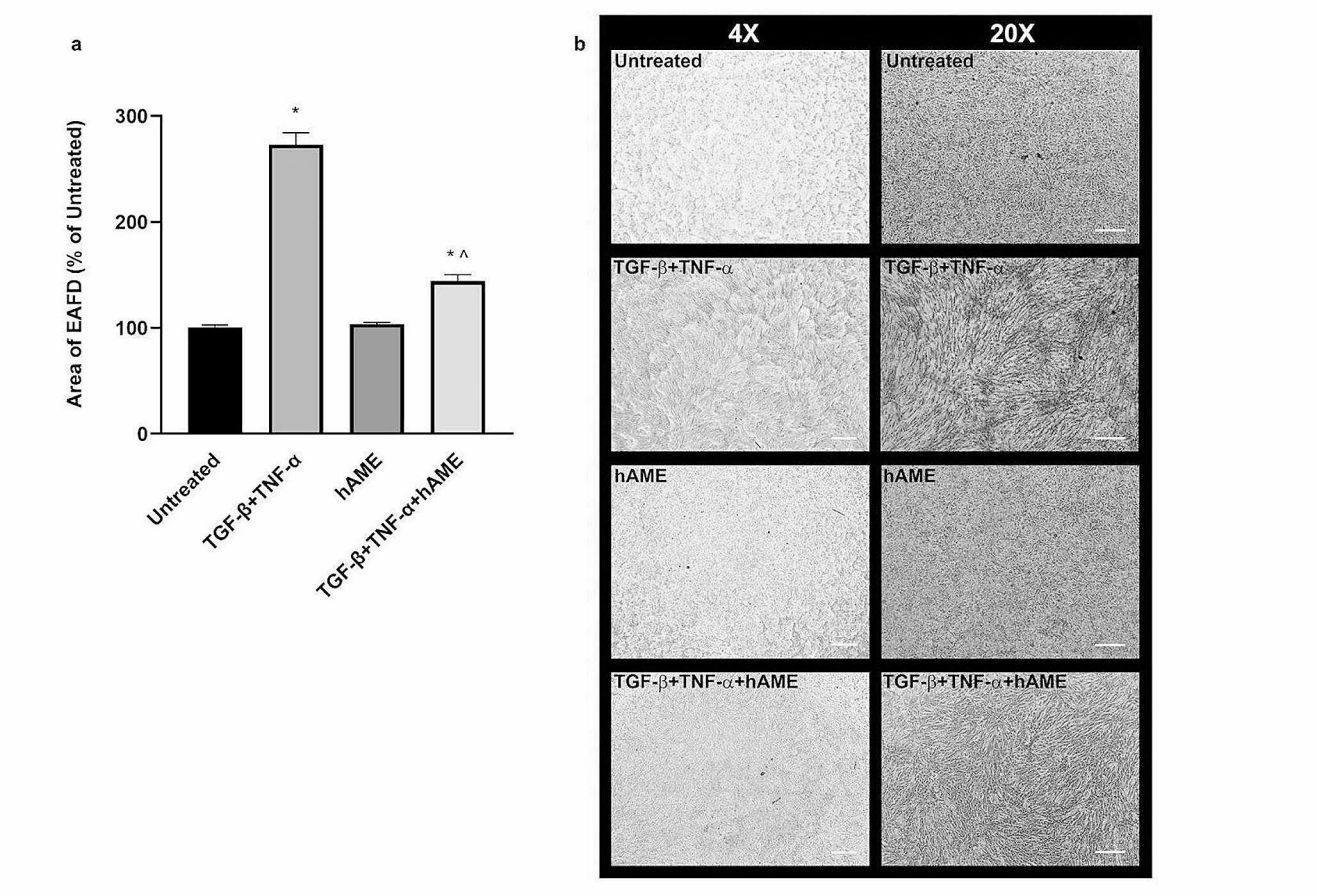
Effect of hAME on EAFD formation in ARPE-19 cells treated with hAME in the presence or not of TNF-α + TGF-β2. (**a**) EAFD area is reported in the graph as % of untreated cells. (**b**) Representative images of EAFD assay (scale bars: 400 and 100 μm, 4X and 20X magnification, respectively). * *p* < 0.05 vs. untreated; ^ *p* < 0.05 vs. hAME

Finally, to evaluate if hAME is able to inhibit EMT-associated fibrotic deposit (EAFD) formation, we used a protocol established by Takahashi et al. [18]. In particular, ARPE-19 cells were treated with TNF-α + TGF-β2, known inducers of EAFD, in the presence or absence of hAME. Results in Fig. 6a-b reveal that cytokines treatment induced an increase of about 180% in the area occupied by EAFDs with respect to the untreated control, and that the hAME was able to reduce this increase to about 45%.

## Discussion

Retina is a highly structured, polarised, and specialised tissue, with cells of different histological origins. Healing of a heavily damaged retina would require the coordinated intervention of many differentiated cell types, most of which are endowed with scarce plasticity and stem potency, to reconstitute a complex tissue architecture [20]. Spontaneous retinal healing has not been extensively studied in humans, however, it appears to have lower possibilities than in other vertebrates, such as fishes or amphibians, especially when cell regeneration should occur [20]. While Müller cells can regenerate and provide different neural cell types to some extent [21], RPE cells have a limited regenerative potential [22, 23], but rather undergo mesenchymal trans-differentiation and subsequent uncontrolled proliferation when exposed to factors from vitreous, thus contributing to PVR [24].

Prompted by the preliminary observation, showing an apparent pro-proliferative effect on ARPE-19 cells of the whole hAM, we produced an extract to assure reproducibility, manageability, and precise dosing for experimental procedures. Our results indicate that hAME was able to increase viability in ARPE-19 cells and to enhance in vitro proliferation. Hillenmeyer et al. [15] found that the exposition of ARPE-19 cells to a fragment 2 × 2 mm of hAM for 3 days, in serum-free medium, did not increase proliferation albeit increasing viability. hAM is used differently in the two studies: as an extract in the former and as a fragment in the latter, and by different experimental protocols. In addition, hAME was able to strongly prevent the reduction of cell viability induced by oxidative stress (H_2_O_2_ or TBPH). Despite the positive effect on cell viability, hAME treatment did not protect cells from apoptosis induced by oxidative stress; this is apparently in contrast with a paper of Krishna et al. [25] in which the whole hAM instead protects the ARPE-19 cells from hyperoxia. However, the experimental setting of these authors was very different in that ARPE-19 cells were grown onto a decellularized whole hAM with the aim of producing a transplantable tissue.

We then looked at the hAME effects on cell migration, EMT marker expression, and EAFD formation, because it was demonstrated that the mesenchymal transition in the RPE cells contributes to retina alterations observed in PVR and diabetic vitreoretinopathy [26]. ZO-1 downregulation, N-Cad upregulation (two well-known markers of epithelial and mesenchymal phenotype [27]), and EAFD formation were induced by ARPE-19 cell exposure to TNF-α and TGF-β2. hAME treatment consistently reduced the effect of cytokines on EAFD formation, prevented the ZO-1 decrease and N-Cad increase at the level of the untreated, respectively. In migration assays, hAME treatment was able to reduce the motility of unstimulated ARPE-19 cells, and of TGF-β1-stimulated cells.

These hAME properties observed in vitro could explain the good outcomes of surgery employing hAM, and support the notion that regeneration could have occurred, while fibrosis could have been prevented, in patients. Of note, in our operative setting [5], complete anatomical continuity was achieved in recurrent macular holes using a patch of hAM, indicative of regeneration, and no scar was observed.

## Conclusions

Although the composition of hAM has been studied for a long time, we do not know much about the versatility of its many properties. Furthermore, the heterogeneity of the hAM (it is known that the composition and properties are different in different districts [28]) could represent both a limitation and an opportunity in several applications and deserve to be investigated, especially before routine clinical use. These findings encourage us to increase the efforts to unravel the potential of this natural product such as hAM, in different forms or compositions, that can be useful for the treatment of retinal pathologies. There is still much to understand about the relationships between the factors contained in the hAME and in the eye microenvironment, in which retinal functioning could be affected by different conditions.

In summary, the results of the present study suggest that the hAM patch used in clinical settings can exert more than simple mechanical support. With this in mind, the extract formulation deserves to be investigated for future use in various eye diseases as a primary or neoadjuvant/adjuvant treatment, offering a new perspective.

## Electronic supplementary material

Below is the link to the electronic supplementary material.

Supplementary Material 1

## Data Availability

Data will be made available on reasonable request.
