# Supplementary figures and images for: Effects of a human amniotic membrane extract on ARPE-19 cells
Source: Mol Biol Rep. 2024 Jun 14;51(1):746. doi: 10.1007/s11033-024-09647-7 (PMC11178654; doi:10.1007/s11033-024-09647-7)

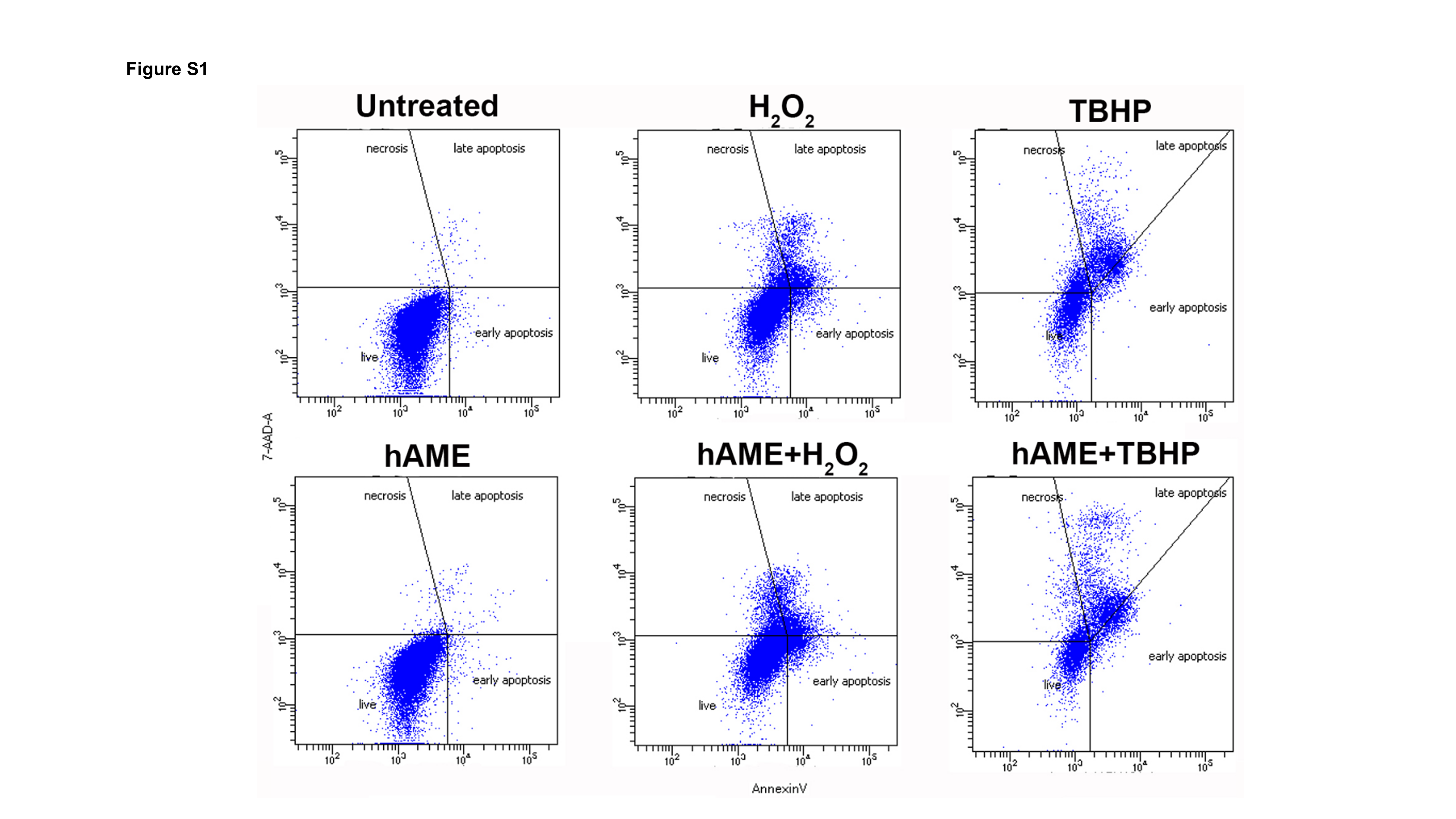

Supplement: Supplementary file 1 — Supplementary Material 1 [file 11033_2024_9647_MOESM1_ESM.tif]
